# Differential associations of urbanicity and income with physical activity in adults in urbanizing China: findings from the population-based China Health and Nutrition Survey 1991-2009

**DOI:** 10.1186/s12966-015-0321-2

**Published:** 2015-12-12

**Authors:** Samantha M. Attard, Annie-Green Howard, Amy H. Herring, Bing Zhang, Shufa Du, Allison E. Aiello, Barry M. Popkin, Penny Gordon-Larsen

**Affiliations:** Department of Nutrition, Gillings School of Global Public Health, University of North Carolina at Chapel Hill, Chapel Hill, NC 27599 USA; University of North Carolina at Chapel Hill, Carolina Population Center CB#8120, 137 East Franklin Street, Chapel Hill, NC 27516-3997 USA; Department of Biostatistics, Gillings School of Global Public Health & School of Medicine, University of North Carolina at Chapel Hill, Chapel Hill, NC 27599 USA; National Institute for Nutrition and Health, Chinese Centers for Disease Control and Prevention, Beijing, 100050 China; Department of Epidemiology, Gillings School of Global Public Health & School of Medicine, University of North Carolina at Chapel Hill, Chapel Hill, NC 27599 USA; School of Medicine, University of North Carolina at Chapel Hill, Chapel Hill, NC 27599 USA

**Keywords:** Urbanization, Epidemiology, Longitudinal, Globalization

## Abstract

**Background:**

High urbanicity and income are risk factors for cardiovascular-related chronic diseases in low- and middle-income countries, perhaps due to low physical activity (PA) in urban, high income areas. Few studies have examined differences in PA over time according to income and urbanicity in a country experiencing rapid urbanization.

**Methods:**

We used data from the China Health and Nutrition Survey, a population-based cohort of Chinese adults (n = 20,083; ages 18-75y) seen a maximum of 7 times from 1991-2009. We used sex-stratified, zero-inflated negative binomial regression models to examine occupational, domestic, leisure, travel, and total PA in Chinese adults according to year, urbanicity, income, and the interactions among urbanicity, income, and year, controlling for age and region of China.

**Results:**

We showed larger mean temporal PA declines for individuals living in relatively low urbanicity areas (1991: 500 MET-hours/week; 2009: 300 MET-hours/week) compared to high urbanicity areas (1991: 200 MET-hours/week; 2009: 125 MET-hours/week). In low urbanicity areas, the association between income and total PA went from negative in 1991 (*p* < 0.05) to positive by 2000 (*p* < 0.05). In relatively high urbanicity areas, the income-PA relationship was positive at all time points and was statistically significant at most time points after 1997 (*p* < 0.05). Leisure PA was the only domain of PA that increased over time, but >95 % of individuals in low urbanicity areas reported zero leisure PA at each time point.

**Conclusions:**

Our findings show changing associations for income and urbanicity with PA over 18 years of urbanization. Total PA was lower for individuals living in more versus less urban areas at all time points. However, these differences narrowed over time, which may relate to increases in individual-level income in less urban areas of China with urbanization. Low-income individuals in higher urbanicity areas are a particularly critical group to target to increase PA in China.

**Electronic supplementary material:**

The online version of this article (doi:10.1186/s12966-015-0321-2) contains supplementary material, which is available to authorized users.

## Background

Greater urbanization and higher socioeconomic status are each risk factors for non-communicable diseases like cardiovascular disease (CVD) in low- and middle- income countries (LMICs) [1]. In LMICs, high urbanicity and income are associated with low levels of physical activity (PA), [1–4] whereas low urbanicity and income are associated with low levels of PA in high-income countries [2, 5–8]. Yet, it remains unclear whether this transition from low PA at high income and urbanicity to low PA at low income and urbanicity unfolds differently by urbanicity and income within a single country over time. Urbanization is often inaccurately captured in the literature with a focus on population density. Urbanization-related changes are broader and include changes to the physical environment, including appearance of paved roads, public transportation, and increased use of labor-saving devices, as well as increases in accessibility of amenities, resulting in less PA as well as shorter bouts of PA [9–11]. These changes occur unevenly over time, depending upon stage of urbanization and income, resulting in gaps in understanding concerning how urbanization relates to inequities in behaviors and health.

The current literature addressing the impact of urbanization on the interplay between urbanicity and income with total PA is limited by the use of cross-sectional samples [2, 7, 8]. As such, we lack insight into how the changing relationships among income, urbanicity, and PA unfold over time. Even the longitudinal studies in the current literature focus on secular trends in PA rather than examining time-varying associations between urbanicity and PA, [2, 9, 12] so it is unclear whether there is a uniform decline in PA in urban and rural areas over time, or whether declines in PA occur differently in rural versus urban areas as they undergo urbanization. Further, although declines in total PA with urbanization are documented in the literature, [2, 9, 12] little is known about which domains of PA (e.g., occupational, domestic, travel, or leisure PA) and which subpopulations have the greatest declines with urbanization. Most studies dichotomize PA outcomes as physically active versus inactive, [13–15] limiting understanding of whether it is participation in, or amount of, PA that changes over time. Furthermore, the literature is populated with studies using classification of urbanicity by population size or density, thereby missing infrastructure, service, social, and amenity sectors that constitute an urban environment and have potential influence on lifestyle behaviors, such as physical activity [16, 17]. Furthermore, the bulk of the literature is based on stable classification of urbanicity, and are thus unable to address temporal changes in infrastructure, social, and amenity sectors elements that relate to changes in PA.

To address these gaps, we used data from Chinese adults (ages 18-75y) seen up to 7 times in the China Health and Nutrition Survey (CHNS) from 1991-2009. We capitalize upon detailed data on urbanization-related changes in infrastructure and service sector as well as domain-specific PA data (e.g., occupational, domestic, travel, and leisure PA) across 18 years of follow-up. We examined whether individual-level income modifies the association between urbanicity and domains of PA over a period of rapid urbanization. We hypothesize that declines in total PA over time occur across all areas, but that the largest declines occur for high-income individuals living in higher urbanicity areas due to urbanicity- and income-related declines in occupational PA over time.

## Methods

The China Health and Nutrition Survey (CHNS) is a longitudinal study with ongoing data collection across 228 communities within 9 provinces of China. Surveys began in 1989, with subsequent exams every 2-4 years, for a total of 9 rounds between 1989 and 2011. The CHNS was designed to provide representation of rural, urban, and suburban areas varying substantially in geography, economic development, public resources, and health indicators [18] and is the only large-scale, longitudinal study of its kind in China. The original exam in 1989 used a multistage, random cluster design in eight provinces (Liaoning, Jiangsu, Shandong, Henan, Hubei, Hunan, Guangxi, and Guizhou) to select a stratified probability sample; a 9^th^ province, Heilongjiang, was added in 1997 using a similar sampling strategy. Essentially two cities (one large and one small city – usually the provincial capital and a lower income city) and four counties (stratified by income: one high, one low, and two middle income counties) were selected in each province. Within cities, two urban and two suburban communities were selected; within counties, one community in the capital city and three rural villages were chosen. Twenty households per community were then selected for participation. All households in the original sample were eligible for inclusion in following surveys and additional households were added in 1997 and in subsequent surveys using identical sampling, primarily to reflect aging of the cohort and urban reconstruction. Over 78 % of households participated in 5-9 surveys and an additional 15 % in 3-4 surveys [18] and there is limited evidence of selectivity in loss to follow up [19]. There was no financial incentive to participate and all participants provided written informed consent.

The study met the standards for the ethical treatment of participants and was approved by the Institutional Review Boards of the University of North Carolina at Chapel Hill and the National Institute for Nutrition and Health, Chinese Center for Disease Control and Prevention. More detailed survey procedures can be found elsewhere [18].

### Sample

The eligible sample for this analysis included 20,374 adults aged 18-75y seen at at least one of the 1991, 1993, 1997, 2000, 2004, 2006, or 2009 exams (64,247 observations [obs]). We excluded 291 individuals who had missing covariate data (n = 183), were pregnant at the time of measurement (n = 106), or had a combination of the two (n = 2), resulting in an analytic sample of 20,083 individuals (62,193 obs; mean number of exams: 4.4). Compared to the 291 individuals excluded due to pregnancy or missing covariate data, the 20,083 in the analytic sample were more likely to be younger, live in less urban areas, and report higher total PA.

#### Outcome variable measurement

At all exam years, participants reported the timing and intensity of occupational and domestic activities using a detailed questionnaire similar to previously validated tools [20–23] but tailored to capture the majority of activities that the Chinese population engages in, including active and inactive transportation modes, occupation, home production, and leisure activity. Starting in 1997, participants additionally reported the types and amount of travel and leisure PA. Participants reported the hours per week spent working in the last year for up to two market-sector jobs and/or income-generating activities at home (e.g., working on a farm, caring for animals, or having a home business), including time spent sitting, standing, walking and lifting heavy loads, which we then categorized as occupational PA according to metabolic equivalents (sitting was not counted as PA) [12]. We categorized time spent preparing food, buying food, doing laundry, and in childcare as domestic PA [12]. Participants reported time spent walking or biking to work (including multimodal trips), which we categorized as travel PA for a measure of total travel (passive travel by car was not counted as PA) [12]. Lastly, we categorized time spent running, gymnastics, dancing, swimming, badminton, ball-sports (e.g. basketball, tennis) or doing martial arts as leisure PA [12]. We assigned metabolic (MET)-equivalent hours using the Compendium of Physical Activities [24, 25] and calculated average hours per week for each of the activity domains (occupational, domestic, travel, and leisure PA). We defined total PA as the sum of the MET-hours for occupational, domestic, travel, and leisure PA.

#### Main exposure variable measurement

Urbanization in China has been rapid and has varied spatially and temporally, with some communities gaining amenities and infrastructure over time, while other communities lose factories or other features that would characterize a more urban environment [16]. Thus, we used detailed individual-level and community-level data to create a multicomponent scale comprised of 12 items that represented infrastructure, economic, and social service domains that define and distinguish the features of urban environments in China [16]. Points were allocated on the basis of presence (or number) of infrastructure or facilities in each of the 12 domains, with a possible range of 0-120 (with a higher score reflecting more urban characteristics across multiple domains). The scale was developed specifically for the CHNS, has high reliability, validity, and temporal stability [16, 17]. We then categorized the resulting scale into year-specific tertiles (low, medium, and high) to reflect the degree of urbanization in each of the CHNS communities over time. We also examined household income, derived from individual and household questionnaires inflated to 2009 Yuan currency in analysis [19]. We include both household income as well as community-level diversity in income from the urbanization scale to allow us to examine the effects of living in a high income household in a high income area, versus a high income household in a more heterogeneous community with variation in income across all households.

#### Covariate measurement

Participants reported age and sex at each time point using standard questionnaires. We entered age as a categorical variable (18-35y, 35-55y, 55-75y) in all models because we observed a non-linear association between age and total PA in initial model testing.

#### Descriptive analysis

We conducted all analyses in Stata 13 (Stata Corp, College Station, TX, USA). In descriptive analyses, we examined changes in median occupational, domestic, travel, leisure, and total PA over time by sex. We tested differences in median sample characteristics over time using Kruskal-Wallis tests. Additionally, we present the proportion of individuals reporting zero time spent in each PA domain and total PA at three time points: 1991, 2000, and 2009, according to tertiles of urbanicity and income and by sex. We tested differences in the proportion of zeros over time for each combination of income and urbanicity level using Chi Squared tests. For all descriptive analysis, we utilized a p value <0.05 as our statistical significance level.

#### Statistical analysis

In many populations, a high proportion of individuals report zero values for many domains of PA, resulting in challenges for examining PA as an outcome in statistical models. A high proportion of zero values results in a skewed distribution for PA, violating normality assumptions inherent in linear regression models. We used a zero-inflated negative binomial model because there are multiple processes that generate zeros (e.g., individuals with highly sedentary occupations report no occupational activity; individuals who are retired are ineligible to participate in occupational activity). We modeled the probability of these excess zeros as well as the mean METs of the remaining population, while allowing for the uncertainty that an individual who reports zero METs could come from either process (A series of Vuong tests [26] indicated that accounting for zero-inflation was necessary, *p* < 0.001). Thus, we used zero-inflated negative binomial models to predict total PA as well as domestic, occupational, travel, and leisure PA in separate models stratified by sex for a total of 10 models. We used a Huber-White/sandwich estimator and clustered standard errors to account for correlation within individuals over time.

We hypothesized that differences in income could impact the association between urbanicity and PA and that these differences could change over time. For example, in earlier years, we hypothesized higher income in higher urban areas would be associated with lower PA, due to a greater number of service occupations in the higher income individuals; whereas in less urban areas higher income may be associated with higher PA due to greater number of hours spent in higher labor occupations. However, in later years, when more areas had experienced at least some urbanization, income differences in PA may be similar at all levels of urbanization. Thus, we tested all two- and three-way interactions among urbanicity, income, and time in our analysis. We used a Wald test to determine whether income by urbanicity interaction terms (including three-way interactions with time) were statistically significant at the *p* < 0.05 level in the rest of our models. These two- and three-way interaction terms were statistically significant for all other models, with the exception of domestic PA for women. We retained the income by urbanicity interaction in this model for consistency with the domestic PA model for men. In models for leisure PA, we did not include interaction terms between urbanicity and income (or the three-way interaction between urbanicity, income, and time) due to a very small proportion of low income, less urban individuals reporting leisure PA, leading to unstable model estimates.

#### Sensitivity analysis

First, because participants age in and out of our sample over the 18 years of follow-up, we have a mixture of adults seen at 1 to 7 time points. Thus, we conducted a complete-case analysis in adults eligible at all 7 time points to determine how income and urbanicity relate to PA domains in the same individuals over time. Second, time spent in sedentary activities may be associated with CVD risk independent of PA [27–29]. We used a zero-inflated negative binomial model to examine time-varying associations between income and urbanicity with leisure sedentary behavior in 2004, 2006, and 2009 (the only three waves where time spent in leisure activities like reading and watching television were reported), to examine changes in sedentary behavior.

## Results

### Descriptive results

From 1991 to 2009, median urbanicity and income increased, while total PA decreased (*p* < 0.05; Table 1). Occupational PA made up the majority of total PA at each time point. The proportion of individuals reporting zero PA increased over time for all domains except for leisure PA with a particularly large increase in reporting of no occupational activity by high urbanicity (Table 2). While mean urbanicity had a steady increase from 1991 to 2009, there was a sharper increase in high income over the same period (Additional file 1: Figure S1).

**Table 1:** Median sample characteristics over time according to sex, China Health and Nutrition Survey 1991-2009^a^

	Men			Women		
	1991	2000	2009	1991	2000	2009
N	4041	4410	4469	4382	4730	4768
Age, years	38.3	43.6	48.2	38.0	43.7	48.9
	(28.0, 51.3)	(32.3, 54.1)	(38.2, 58.8)	(28.1, 50.8)	(33.3, 53.9)	(38.9, 59.0)
Income, 1000 Yuan^b^	8.6	10.4	19.5	8.6	10.3	17.9
	(13.0, 19.7)	(18.7, 29.9)	(34.9, 58.8)	(13.0, 19.5)	(18.5, 30.0)	(33.0, 56.7)
Urbanicity^c^	32.1	43.0	50.8	32.3	43.5	51.1
	(44.5, 61.0)	(55.8, 76.8)	(64.2, 85.1)	(44.9, 61.0)	(57.4, 76.9)	(65.6, 85.4)
Total PA^d^						
MET-hours/week	336.0	239.9	160.0	401.7	243.9	143.2
	(168.0, 576.0)	(106.0, 385.5)	(73.0, 301.5)	(192.0, 651.1)	(94.0, 420.5)	(61.7, 304.4)
Hours/week	70.0	56.0	50.5	89.3	61.6	51.2
	(51.0, 102.0)	(40.7, 83.3)	(21.0, 77.8)	(62.0, 125.5)	(35.8, 92.7)	(23.3, 80.0)
Occupational PA^d^						
MET-hours/week	304.0	224.0	138.0	330.0	204.0	80.0
	(132.0, 558.0)	(80.0, 366.0)	(36.0, 280.0)	(106.0, 582.0)	(42.0, 366.0)	(0.0, 240.0)
Hours/week	60.0	54.0	49.0	60.0	48.5	40.0
	(48.0, 96.0)	(40.0, 82.0)	(39.0, 80.0)	(48.0, 98.0)	(37.0, 77.0)	(8.0, 63.0)
Domestic PA^d^						
MET-hours/week	1.7	0.0	2.8	49.7	32.0	38.1
	(0.0, 19.3)	(0.0, 13.1)	(0.0, 18.7)	(23.6, 78.9)	(15.8, 48.8)	(22.4, 58.3)
Hours/week	1.0	0.0	1.2	21.0	14.0	16.3
	(0.0, 8.2)	(0.0, 5.8)	(0,0, 8.2)	(10.3, 32.8)	(7.0, 21.0)	(9.3, 24.5)
Leisure PA^d^						
MET-hours/week	--	0.0	0.0	--	0.0	0.0
		(0.0, 0.0)	(0.0, 0.0)		(0.0, 0.0)	(0.0, 0.0)
Hours/week	--	0.0	0.0	--	0.0	0.0
		(0.0, 0.0)	(0.0, 0.0)		(0.0, 0.0)	(0.0, 0.0)
Travel PA^d^						
MET-hours/week	--	5.0	0.0	--	4.7	0.0
		(0.0, 12.5)	(0.0, 5.0)		(0.0, 10.8)	(0.0, 5.0)
Hours/week	--	2.5	0.8	--	2.5	1.7
		(1.3, 5.0)	(0.0, 2.5)		(1.3, 5.0)	(0.0, 2.5)

^a^All cells are shown as median (25^th^ percentile, 75^th^ percentile). Kruskal-Wallis tests for difference in median values over time were statistically significant at the p < 0.05 level for all characteristics in men and women

^b^Household income derived inflated to 2009 Yuan values

^c^Urbanicity defined by a multicomponent urbanicity scale with possible score of 0-120 points

^d^PA values derived from self-reported questionnaires

Abbreviations: *PA* physical activity

**Table 2:** Percentage of individuals reporting no PA, China Health and Nutrition Survey 1991-2009^a,b^

	Men			Women		
	1991	2000	2009	1991	2000	2009
Total PA (proportion of sample reporting zero)						
Low Urbanicity						
Low Income	0.5 (0.3)*	2.7 (0.6)*	7.6 (1.1)*	0.7 (0.3)*	1.0 (0.4)*	2.6 (0.6)*
Med Income	0.2 (0.2)*	1.8 (0.6)*	3.1 (0.8)*	0.6 (0.4)*	1.6 (0.6)*	2.5 (0.7)*
High Income	1.4 (0.6)	0.8 (0.5)*	4.5 (1.0)*	0.8 (0.5)*	1.1 (0.5)*	3.2 (0.9)*
Med Urbanicity						
Low Income	2.3 (0.7)*	5.8 (1.1)*	12.4 (1.5)*	0.7 (0.4)*	5.2 (1.0)*	4.3 (0.9)*
Med Income	3.1 (0.9)*	3.3 (0.8)*	7.6 (1.2)*	1.8 (0.6)	3.5 (0.8)	3.2 (0.8)
High Income	1.7 (0.6)*	3.5 (0.8)*	5.4 (1.0)*	0.6 (0.3)	2.1 (0.6)	2.1 (0.6)
High Urbanicity						
Low Income	4.2 (1.2)*	11.4 (1.7)*	16.9 (2.0)*	0.9 (0.5)*	6.5 (1.2)*	2.8 (0.8)*
Med Income	2.7 (0.7)*	8.7 (1.3)*	11.5 (1.4)*	2.3 (0.6)	3.5 (0.8)	2.0 (0.6)
High Income	4.2 (1.2)*	11.4 (1.7)*	16.9 (2.0)*	0.9 (0.5)	6.5 (1.2)	2.8 (0.8)
Occupational PA (proportion of sample reporting zero)						
Low Urbanicity						
Low Income	0.7 (0.3)*	5.0 (0.8)*	13.0 (1.4)*	1.3 (0.5)*	8.7 (1.1)*	20.7 (1.6)*
Med Income	0.7 (0.4)*	3.7 (0.9)*	7.3 (1.2)*	0.9 (0.4)*	6.2 (1.1)*	14.5 (1.6)*
High Income	1.7 (0.7)	4.9 (1.1)*	6.8 (1.2)*	2.4 (0.8)*	3.7 (1.0)*	13.1 (1.7)*
Med Urbanicity						
Low Income	3.6 (0.9)*	15.9 (1.7)*	29.3 (2.1)*	10.1 (1.3)*	26.3 (2.0)*	37.3 (2.1)*
Med Income	3.6 (1.0)*	10.6 (1.4)*	18.9 (1.8)*	6.6 (1.2)*	19.6 (1.7)*	30.8 (2.1)*
High Income	2.3 (0.7)*	8.5 (1.3)*	16.0 (1.6)*	4.3 (0.9)*	13.9 (1.5)*	28.7 (2.0)*
High Urbanicity						
Low Income	9.0 (1.7)*	46.4 (2.7)*	55.3 (2.7)*	25.6 (2.5)*	61.3 (2.4)*	69.4 (2.2)*
Med Income	5.1 (1.0)*	31.9 (2.1)*	35.7 (2.1)*	11.5 (1.3)*	39.2 (2.1)*	56.5 (2.1)*
High Income	5.6 (1.0)	18.0 (1.5)*	28.2 (1.9)*	12.3 (1.4)*	33.5 (1.8)*	46.7 (2.0)*
Domestic PA (proportion of sample reporting zero)						
Low Urbanicity						
Low Income	50.3 (2.1)*	63.8 (1.9)*	45.9 (2.1)*	6.6 (1.0)*	11.9 (1.2)*	5.8 (0.9)*
Med Income	55.7 (2.4)*	56.1 (2.3)*	48.7 (2.2)*	7.7 (1.2)*	11.1 (1.4)*	5.4 (1.0)*
High Income	52.8 (2.7)*	54.6 (2.6)*	51.9 (2.4)*	12.8 (1.7)*	11.7 (1.7)*	7.5 (1.3)*
Med Urbanicity						
Low Income	45.4 (2.3)*	63.4 (2.2)*	45.7 (2.2)*	3.9 (0.8)*	13.3 (1.5)*	6.5 (1.0)*
Med Income	51.2 (2.6)*	53.4 (2.2)*	52.1 (2.3)*	8.2 (1.3)*	12.1 (1.4)*	6.0 (1.1)*
High Income	47.4 (2.3)*	52.4 (2.3)*	52.1 (2.2)*	12.1 (1.4)*	9.8 (1.3)*	7.9 (1.2)*
High Urbanicity						
Low Income	37.4 (2.9)*	47.3 (2.7)*	40.1 (2.6)*	4.4 (1.2)*	15.7 (1.8)*	7.8 (1.3)*
Med Income	36.7 (2.1)*	47.2 (2.2)*	44.9 (2.2)*	7.7 (1.1)*	13.8 (1.5)*	8.1 (1.2)*
High Income	41.4 (2.1)*	45.2 (2.0)*	42.0 (2.1)	8.2 (1.2)*	14.8 (1.4)*	8.3 (1.1)*
Leisure PA (proportion of sample reporting zero)						
Low Urbanicity						
Low Income		94.7 (0.9)*	98.5 (0.5)*		99.0 (0.4)	99.1 (0.4
Med Income		94.5 (1.1)*	98.0 (0.6)*		97.1 (0.8)	99.0 (0.4)
High Income		91.4 (1.5)*	95.3 (1.0)*		98.9 (0.5)	97.1 (0.8)
Med Urbanicity						
Low Income		91.4 (1.3)	93.9 (1.1)		97.3 (0.7)	94.3 (1.0)
Med Income		87.5 (1.5)	90.5 (1.3)		93.3 (1.0)*	91.9 (1.2)*
High Income		85.3 (1.6)	85.5 (1.5)		94.9 (1.0)*	91.5 (1.2)*
High Urbanicity						
Low Income		84.7 (2.0)	88.3 (1.7)		92.5 (1.3)*	88.7 (1.5)*
Med Income		80.0 (1.8)*	78.9 (1.8)*		88.7 (1.4)*	81.4 (1.7)*
High Income		77.3 (1.7)*	74.4 (1.8)*		88.1 (1.3)*	79.9 (1.6)*
Active Commuting PA (proportion of sample reporting zero)						
Low Urbanicity						
Low Income		44.1 (1.9)*	43.3 (2.0)*		48.9 (1.9)*	50.5 (2.0)*
Med Income		34.9 (2.2)*	49.9 (2.2)*		35.6 (2.2)*	50.0 (2.2)*
High Income		37.3 (2.5)*	54.7 (2.4)*		32.5 (2.4)*	55.5 (2.5)*
Med Urbanicity						
Low Income		54.0 (2.3)*	62.6 (2.2)*		53.3 (2.3)*	63.4 (2.0)*
Med Income		42.6 (2.2)*	56.8 (2.2)*		39.8 (2.0)*	57.3 (2.2)*
High Income		43.7 (2.3)*	60.4 (2.2)*		42.7 (2.1)*	56.4 (2.2)*
High Urbanicity						
Low Income		42.5 (2.7)*	73.1 (2.4)*		42.1 (2.5)*	85.5 (1.7)*
Med Income		41.9 (2.2)*	63.3 (2.1)*		36.1 (2.1)*	74.2 (1.9)*
High Income		39.5 (2.0)*	61.7 (2.0)*		31.9 (1.8)*	67.4 (1.9)*

^a^Cells represent percent (standard error)

^b^Urbanicity separated into year-specific tertiles (low, medium, and high urbanicity). Household income is separated into year-specific tertiles (low, medium, and high urbanicity)

*Statistically significant difference (*p* < 0.05) in the proportion of individuals reporting zero PA at a specific income or urbanicity level using a Chi-Squared test

Abbreviations: *PA*, physical activity

### Adjusted model results

**Fig. 1:**
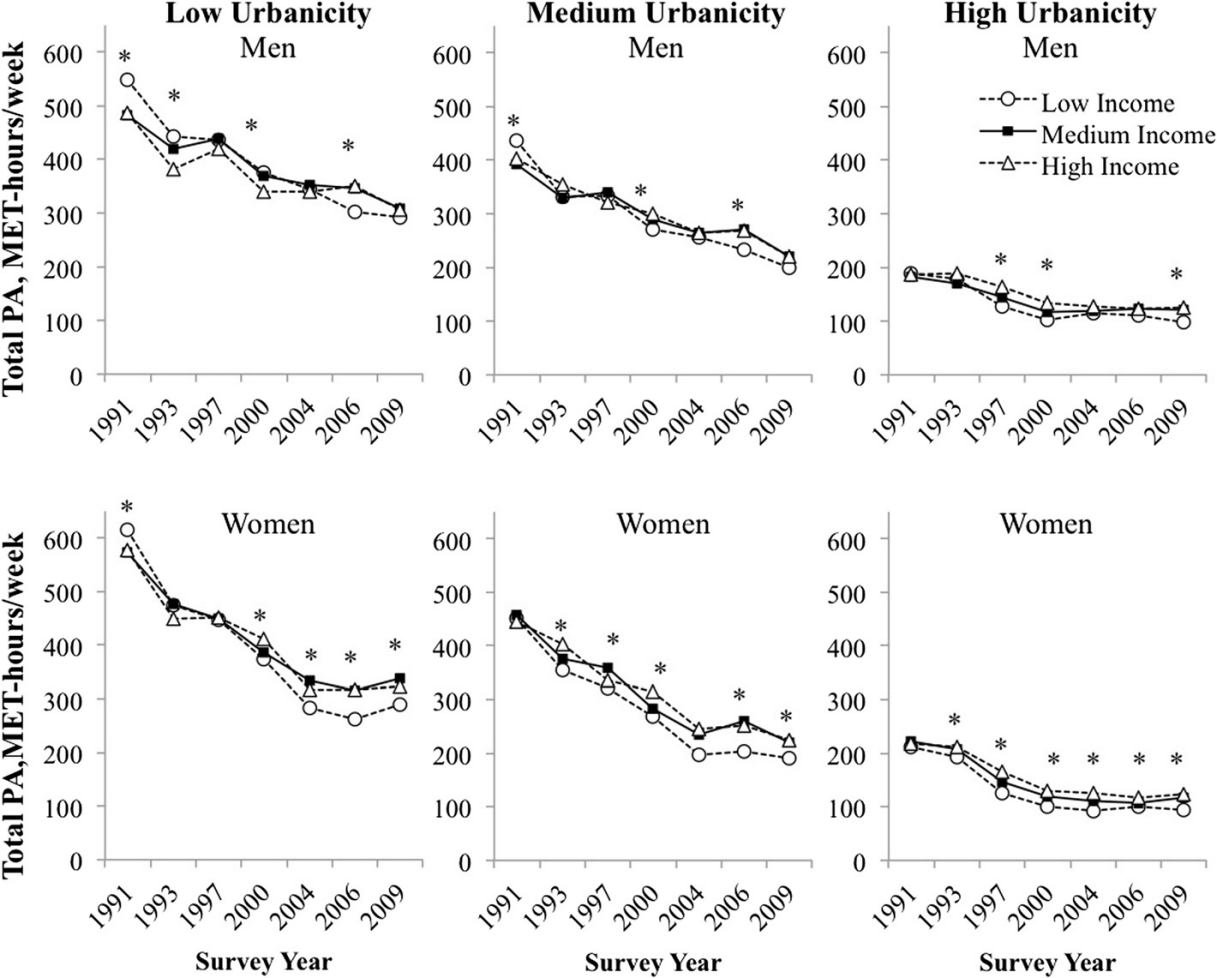
Predicted total PA according to urbanicity and income levels, China Health and Nutrition Survey 1991-2009^a^. ^a^Total physical activity predicted from sex-stratified, zero-inflated negative binomial models according to urbanicity (year-specific tertiles of low, medium, and high urbanicity) and income (year-specific tertiles of low, medium, and high income). Main exposure variables were year, urbanicity, income, the interaction between urbanicity and income, and the interaction between urbanicity, income and year. Models additionally control for region of China (North, Central, South) and age (ages 18-35y, 35-55y, 55-75y). Stars denote a statistically significant difference in mean PA for high versus low income at the *p* < 0.05 level for at each year. Abbreviations: PA, physical activity

Using model-adjusted predictions for mean total PA from 1991 to 2009, adjusted mean total PA declined over time among individuals at all urbanicity and income levels (Fig. 1; beta coefficients in Additional file 1: Tables S1 and S2). The largest declines in mean total PA occurred in low urbanicity areas [from 500 (1991) to 300 (2009) MET-hours/week in men and from 550 to 200 MET-hours/week for women over the same period). There was a reversal by income with lower total PA for individuals of high income in less urbanicity areas in 1991 and 1993, but then switched to higher total PA after 2000. In contrast total PA was higher for individuals of high income in more urban areas at all time points. Thus, income differences widened over the study period.

**Fig. 2:**
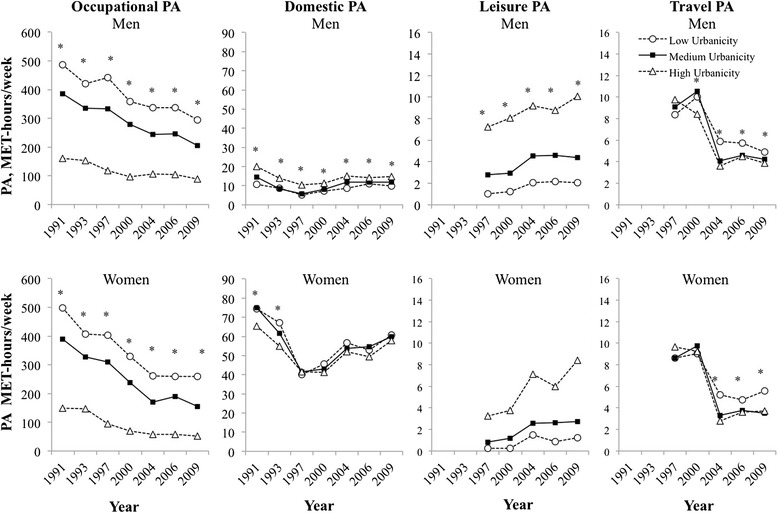
Predicted occupational, domestic, travel, and leisure PA, China Health and Nutrition Survey 1991-2009^a^. ^a^Occupational, domestic, leisure, and travel PA in men (top row) and women (bottom row) predicted from sex-stratified, zero-inflated negative binomial models according to urbanicity (year-specific tertiles of low, medium, and high urbanicity) at medium income. Main exposure variables were year, urbanicity, income, the interaction between urbanicity and income, and the interaction between urbanicity, income and year. Models additionally control for region of China (North, Central, South) and age (ages 18-35y, 35-55y, 55-75y). Abbreviations: PA, physical activity

From 1991 to 2009, occupational PA declined more among individuals of medium income living in relatively low urbanicity areas in comparison to individuals living in relatively high urbanicity areas (Fig. 2, beta coefficients shown in Additional file 1: Tables S1 and S2). Domestic PA was higher in women versus men at all years. Whereas, there was an increase in leisure PA at all urbanicity levels, regardless of income level, although increases were highest in the high urbanicity areas. Travel PA declined from 1997 to 2009 for individuals at all urbanicity levels, with larger declines occurring for individuals living in low versus high urbanicity areas. Additional results by urbanicity and income for each PA domain are shown in Additional file 1: Figures S2-S5 and suggest higher occupational, travel, and leisure PA among high versus low income individuals.

### Sensitivity analyses

We conducted two sensitivity analyses. First, we restricted our sample to individuals included at all 7 exams to determine if there were differences in our complete cases versus the central analyses which included individuals missing at some exams. We observed similar patterns in PA over time, with total PA declining most for individuals in low versus high urbanicity areas, and with differences in PA by income widening over time among individuals living in high urbanicity areas. Second, we examined adjusted mean leisure sedentary behavior by income and urbanicity in 2004, 2006, and 2009, we found that leisure sedentary behavior increased over time for individuals across all levels of urbanicity and income, and that individuals living in high urbanicity areas had higher leisure sedentary behavior than individuals in low urbanicity areas (Additional file 1: Figure S6).

## Discussion

Over the past two decades of urbanization in China, there have been uneven changes in environments and behaviors across initially urban and initially rural areas. Our findings suggest that declines in PA were particularly substantial in less urban areas. In more urban areas, PA was low to begin with and remained stable over time, resulting in a convergence of activity level across rural and urban areas. Yet we also found an increase in PA in some segments of society [e.g., higher (versus lower) income individuals had higher occupational PA across all urbanicity levels; high income individuals also increased their leisure PA in more urban areas]. Changes in occupational PA were largely responsible for the observed declines in total PA for individuals living in less urban areas. Thus, although PA declined with urbanization, individuals living in more urban areas had some replacement of declining occupational PA with increasing leisure PA. However, individuals living in less urban areas did not have an increase in leisure PA that was sufficient to counteract their decline in occupational PA.

Understanding the correlates of longitudinal trends in PA is of great interest to researchers given the increasing prevalence of lifestyle-related cardiometabolic diseases in LMICs [30]. Previous studies reported sharp declines in total PA for Chinese adults from 1991 to 2009, [12, 25] but those studies did not examine whether declines in PA differed among individuals living in rural versus urban areas undergoing urbanization. There are some cross-sectional studies that report lower PA among individuals living in more versus less urbanized areas, [7, 15] but these studies were unable to examine time-varying differences in PA by levels of urbanicity. Thus, whether individuals living in more versus less urbanized areas of an urbanizing country experience different changes in PA over time has not been addressed. In our study, we found larger declines in PA for individuals living in less versus more urban areas with urbanization, likely due to the proliferation of labor-saving devices occurring first in highly urban areas and later spreading to less urbanized areas. These declines translated to an increasing similarity in PA across more and less urban areas over time. Our findings add to a growing body of work suggesting that urbanization-related changes in lifestyle behaviors and health risk extend beyond individuals living in highly urbanized centers to those living in less urban areas [31–33].

We hypothesized initially lower PA for individuals living in highly urbanized areas and with higher income with declines in PA over time for individuals in highly urbanized areas and with higher income. We found income-related disparities in PA that highlight a transition from a pattern of PA traditionally seen in LMICs (higher PA for individuals with lower income [2, 7, 8]) to a pattern seen in most high income countries (higher PA for individuals with higher income [3, 4]). This transition was particularly apparent among individuals in highly urbanized areas, where we observed higher occupational, leisure, and travel PA for individuals of high versus low income. We would have expected lower income individuals to have higher occupational PA if lower income signified a manual labor occupation; however, we observed higher occupational PA for individuals of higher income, possibly because individuals with higher income are working more hours or have multiple forms of employment. Leisure PA, on the other hand increased over time for all income groups, but increased most for individuals of high versus low income. We found that individuals of higher income were more likely to have gym memberships, which may explain the association between income and higher leisure PA. While these differences in leisure PA by income were not large, it is possible that further increases in leisure PA could potentially offset the urbanization-related declines in other PA domains. In less urban areas, differences in PA by income were not as large as those we observed in more urban areas. However, having higher versus lower income was associated with higher total PA in 2009 even in less urban areas. Our findings show differential urbanization-related associations between PA with urbanicity and income. Low income individuals living in more urban areas had the lowest observed PA and thus may be a particularly important group to target in interventions to increase PA.

Using our zero-inflated negative binomial to deal with multiple processes that generate zero values in PA reporting, we found declines in occupational PA in more versus less urban areas, with steeper declines in more urban areas. In higher urbanicity areas, declines in participation in occupational PA may relate to shifts from manual-based occupational activity to service-based industries [9]. In lower urbanicity areas, declines in mean occupational PA may relate to technological advances and influx of labor-saving devices that reduce the amount of occupational PA needed [9, 34]. Thus, it is possible that urbanization influences participation in certain domains of PA as well as the amount of PA.

We observed a large decline in occupational PA from 1991 to 2009, particularly for individuals living in less urbanized areas, likely because improved technology and changing occupations with urbanization lead to more sedentary, service-based occupations [9, 35, 36]. Domestic PA increased slightly between 1991 and 2000 before declining between 2000 and 2009. Like occupational PA, domestic PA declines are likely due to improved technology and more women entering the workforce [9, 35]. We suspect that this pattern relates to the introduction of laundry machines and laundromats first in higher income areas of China in the 1990s and then in lower income areas of China after 2000, causing a shift in laundry-related energy expenditure due to labor saving devices. We also saw a decline in time spent in food shopping, which may relate to the influx of modern markets and away-from-home eating options along with increases in accessibility of amenities occurring unevenly with urbanization, which resulted in uneven patterns of declines in domestic PA. Active commuting PA declined more for individuals living in higher versus lower urbanicity areas, such that commuting PA was higher in 1997 and lower in 2009 for individuals in higher versus lower urbanicity areas. The change we observed in the direction of the association between urbanicity and commuting PA shows a transition from a pattern of commuting PA traditionally observed in LMICs [13] to a pattern similar to that found in high-income countries [35, 37, 38]. We observed increasing leisure PA over time particularly for individuals living in higher urbanicity areas and among individuals with high income. Similar to our findings, leisure PA is primarily observed in highly urbanized countries [2, 10, 37]. We found that higher versus lower income was associated with higher occupational and leisure PA at all urbanicity levels at most time points, while higher versus lower income was associated with higher travel PA only in highly urbanized areas. Together, our findings suggest that relatively stable total PA over time among individuals living in higher urbanicity areas can be attributed to small decreases in occupational PA accompanied by increases in leisure PA over time. Among individuals living in lower urbanicity areas, we found large temporal declines in total PA due to declining occupational PA over time without a commensurate increase in leisure or travel PA.

There is great interest in identifying which PA domains hold most promise as intervention targets in LMICs because strategies used in high income countries may not be feasible or culturally appropriate [4]. Though declines in occupational PA are largely responsible for declines in total PA in LMICs, increasing occupational PA may not be a feasible intervention target because of the shift from manual jobs to service-based industries that occurs with urbanization [2, 30]. Similarly, declines in domestic PA over time may be related to technological advances and thus, domestic PA may not be a viable domain for intervention. Opinions differ on whether leisure PA is sufficient to address the global rise in physical inactivity, [35, 37] but our findings suggest that leisure PA is playing an increasingly important role in PA for Chinese adults, particularly for individuals living in higher urbanicity areas and with higher income. Thus, as occupational PA continues to decrease among individuals in lower urbanicity areas, early adoption of leisure PA in lower urbanicity areas may be a critical strategy for preventing obesity and CVD risk. In our sample, adjusted mean total PA was higher than the 11.25-22.5 MET-hours/week of PA recommended in the World Health Organization Physical Activity Guidelines, [11] primarily due to occupational PA. Because it is likely that occupational PA will continue to decline with urbanization, while participation in all domains of PA is decreasing, it is likely that the proportion of individuals meeting PA recommendations will decline in the coming years. In a sensitivity analysis, we found that mean leisure sedentary behavior increased for individuals across all levels of urbanicity and income between 2004 and 2009. Together with declines in total PA, our observed increases in leisure sedentary behavior highlight the increasingly unhealthy activity behaviors in Chinese adults experiencing urbanization.

Our work advances the understanding of PA transitions in an urbanizing country. An important limitation is that our PA data is based on self-report, which is typical for large, population-based studies. Though accelerometer data is a more objective measure of PA, this technology is relatively new, and was not available in the early 1990s when we began following this cohort.

However, because under- or over-reporting is unlikely to differ within the same individual over time, it is not likely to have affected our parameter estimates for PA over time. In addition, our questionnaire does not provide time-use data though we do capture a wide range of culturally-appropriate activities. Thus, we are unable to fully account for all energy expended in the course of the day. In spite of this limitation, our work has several strengths. First, the data from the CHNS is one of the only longitudinal, population-based studies in a developing country such as China, with data on individual behaviors and community attributes over 20 years of rapid environmental change. We used a multidimensional, time-varying measure of urbanicity that captured urbanization-related changes in infrastructure, social services, and amenities over time [16] as well as rapid technological and demographic changes occurring in China over our study period [34]. With our comprehensive measures of urbanicity and PA and a sophisticated modeling technique, we examined different domains of PA across demographic, socioeconomic, and environmental characteristics finding that income-related disparities in PA increased over a period of urbanization, and highlighting the importance of strategies to increase PA in low income individuals living in less urban areas.

## Conclusion

In summary, our findings suggest that urbanization-related declines in PA in China are common even in less urbanized areas. We also found declines in occupational PA for individuals at all levels of urbanicity, but increases in leisure PA only among individuals in more (but not less) urban area and in individuals of high (rather than low) income. Thus, as China continues to urbanize, efforts to increase leisure PA will be particularly critical for individuals living in less urban areas. In addition, we found widening income disparities in PA over time with disproportionate declines in PA for low income individuals residing in less urban areas, suggesting a particular need to address these differential declines in PA early in the urbanization process.

## Additional file

Additional file 1: Figure S1. Period-specific mean urbanicity and income, China Health and Nutrition Survey 1991-2009^a,b^.** Table S1.** Beta coefficients from zero-inflated negative binomial models in men^a^. **Table S2.** Beta coefficients from zero-inflated negative binomial models in women^a^. **Figure S2.** Predicted mean occupational PA, China Health and Nutrition Survey 1991-2009^a^. **Figure S3.** Predicted domestic PA, China Health and Nutrition Survey 1991-2009^a^. **Figure S4.** Predicted mean travel PA, China Health and Nutrition Survey 1991-2009^a^. **Figure S5.** Predicted mean leisure PA, China Health and Nutrition Survey 1991-2009^a^. **Figure S6.** Predicted mean sedentary behavior^a^. (DOCX 1023 kb)

## Abbreviations

CHNS: China Health and Nutrition Survey
CVD: Cardiovascular disease
LMICs: Low- and middle-income countries
PA: Physical activity
