# Supplementary material for: Differential associations of urbanicity and income with physical activity in adults in urbanizing China: findings from the population-based China Health and Nutrition Survey 1991-2009
Source: Int J Behav Nutr Phys Act. 2015 Dec 12;12:152. doi: 10.1186/s12966-015-0321-2 (PMC4676871; doi:10.1186/s12966-015-0321-2)
Supplement: Additional file 1: Figure S1. — Period-specific mean urbanicity and income, China Health and Nutrition Survey 1991-2009a,b. Table S1. Beta coefficients from zero-inflated negative binomial models in mena. Table S2. Beta coefficients from zero-inflated negative binomial models in womena. Figure S2. Predicted mean occupational PA, China Health and Nutrition Survey 1991-2009a. Figure S3. Predicted domestic PA, China Health and Nutrition Survey 1991-2009a. Figure S4. Predicted mean travel PA, China Health and Nutrition Survey 1991-2009a. Figure S5. Predicted mean leisure PA, China Health and Nutrition Survey 1991-2009a. Figure S6. Predicted mean sedentary behaviora. (DOCX 1023 kb) [file 12966_2015_321_MOESM1_ESM.docx]

**Supplemental Figure 1. Period-specific mean urbanicity and income, China Health and Nutrition Survey 1991-2009^a,b^**

**
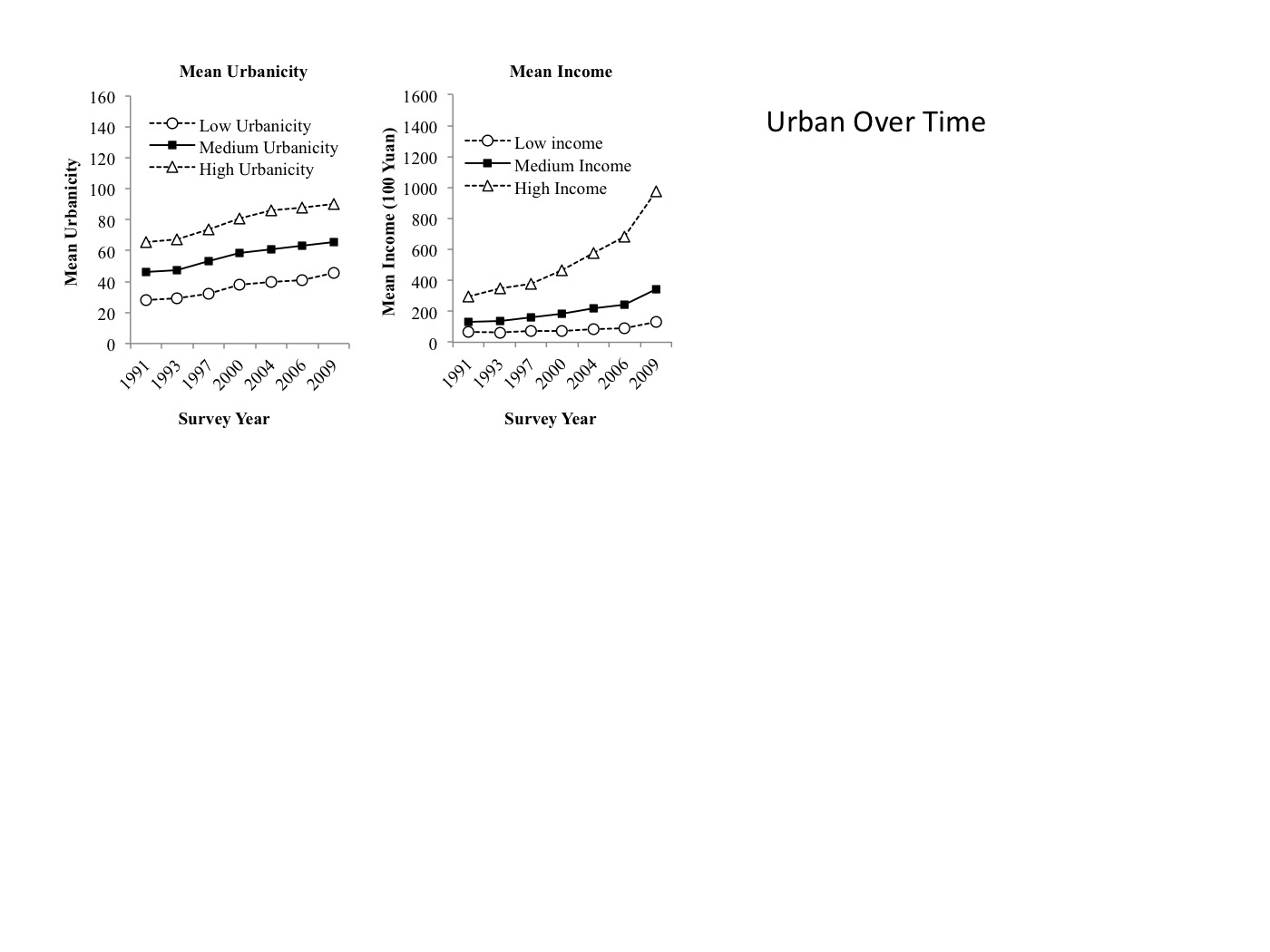
**

^a^Using individual and community-level surveys, a multicomponent urbanicity scale was created to represent the infrastructure, economic, and social service domains of the urban environment.[18] The scale has a possible score of 0-120 points, with a higher score representing more urban characteristics. The urbanicity scale was separated into year-specific tertiles: low, medium, and high urbanicity.

**^b^**Income was derived from household and individual questionnaires and was inflated to 2009 Yuan currency in analysis for comparability over time. We defined year-specific tertiles of income: low, medium, and high income.

**Supplemental Table 1. Beta coefficients from zero-inflated negative binomial models in men^a^**

|  | **Total PA** | **Occupational PA** | **Domestic PA** | **Travel PA** | **Leisure PA** |
| --- | --- | --- | --- | --- | --- |
| Negative Binomial Parameter Estimates | |  |  |  |  |
| Low Urb^b^ | ref | ref | ref | ref | ref |
| Medium Urb^b^ | -0.21 (0.03) | -0.21 (0.03) | -0.10 (0.13) | 0.02 (0.07) | 0.24 (0.11) |
| High Urb^b^ | -1.03 (0.04) | -1.10 (0.04) | -0.04 (0.14) | 0.40 (0.09) | 0.43 (0.11) |
| Low Inc^c^ | ref | ref | ref | ref | ref |
| Medium Inc^c^ | -0.13 (0.02) | -0.11 (0.02) | -0.42 (0.14) | 0.07 (0.07) | 0.02 (0.10) |
| High Inc^c^ | -0.13 (0.03) | -0.12 (0.03) | -0.40 (0.15) | 0.11 (0.08) | 0.03 (0.09) |
| Med UrbXMed Inc^b,c^ | 0.01 (0.04) | -0.01 (0.04) | 0.33 (0.20) | 0.13 (0.11) | 0.00 (0.00) |
| Med UrbXHigh Inc^b,c^ | 0.03 (0.05) | 0.01 (0.05) | 0.39 (0.21) | 0.22 (0.12) | 0.00 (0.00) |
| High UrbXMed Inc^b,c^ | 0.07 (0.05) | 0.04 (0.05) | 0.43 (0.19) | -0.08 (0.12) | 0.00 (0.00) |
| High UrbXHigh Inc^b,c^ | 0.09 (0.05) | 0.07 (0.05) | 0.26 (0.20) | -0.11 (0.12) | 0.00 (0.00) |
| 1991 | ref | ref | ref | -- | -- |
| 1993 | -0.20 (0.02) | -0.20 (0.02) | -0.17 (0.16) | -- | -- |
| 1997 | -0.21 (0.02) | -0.19 (0.02) | -0.78 (0.13) | ref | ref |
| 2000 | -0.36 (0.02) | -0.35 (0.02) | -0.76 (0.13) | 0.12 (0.07) | 0.25 (0.16) |
| 2004 | -0.46 (0.03) | -0.43 (0.03) | -0.51 (0.13) | -0.04 (0.08) | 1.22 (0.27) |
| 2006 | -0.58 (0.03) | -0.52 (0.03) | -0.56 (0.11) | -0.11 (0.07) | 1.05 (0.21) |
| 2009 | -0.58 (0.04) | -0.58 (0.04) | -0.54 (0.11) | -0.11 (0.06) | 1.27 (0.23) |
| Med UrbX1993^b^ | -0.06 (0.04) | -0.05 (0.04) | -0.06 (0.24) | -- | -- |
| Med UrbX1997^b^ | -0.03 (0.04) | 0.01 (0.04) | 0.29 (0.20) | ref | ref |
| Med UrbX2000 ^b^ | -0.08 (0.04) | -0.03 (0.04) | 0.58 (0.20) | 0.27 (0.13) | -0.14 (0.18) |
| Med UrbX2004 ^b^ | -0.07 (0.06) | -0.01 (0.06) | 0.22 (0.18) | -0.34 (0.11) | -0.24 (0.24) |
| Med UrbX2006 ^b^ | -0.03 (0.06) | 0.03 (0.06) | 0.23 (0.17) | -0.15 (0.12) | -0.28 (0.21) |
| Med UrbX2009 ^b^ | -0.12 (0.06) | -0.01 (0.06) | 0.30 (0.16) | -0.36 (0.10) | -0.28 (0.22) |
| High UrbX1993 ^b^ | 0.17 (0.05) | 0.19 (0.05) | 0.10 (0.21) | -- | -- |
| High UrbX1997 ^b^ | -0.13 (0.06) | 0.10 (0.06) | 0.27 (0.18) | -0.20 (0.12) | ref |
| High UrbX2000 ^b^ | -0.19 (0.06) | 0.11 (0.06) | 0.38 (0.20) | -0.79 (0.17) | -0.09 (0.16) |
| High UrbX2004 ^b^ | -0.02 (0.08) | 0.33 (0.07) | 0.34 (0.19) | -0.72 (0.14) | -0.51 (0.23) |
| High UrbX2006 ^b^ | 0.08 (0.08) | 0.46 (0.08) | 0.54 (0.18) | -0.44 (0.13) | -0.61 (0.20) |
| High UrbX2009 ^b^ | 0.06 (0.07) | 0.45 (0.07) | 0.45 (0.17) | -0.20 (0.12) | -0.43 (0.20) |
| Med IncX1993^c^ | 0.07 (0.04) | 0.05 (0.03) | 0.24 (0.25) | -- | -- |
| Med IncX1997 ^c^ | 0.12 (0.03) | 0.11 (0.03) | 0.30 (0.20) | ref | ref |
| Med IncX2000 ^c^ | 0.11 (0.04) | 0.08 (0.04) | 0.49 (0.19) | 0.08 (0.11) | 0.04 (0.16) |
| Med IncX2004 ^c^ | 0.15 (0.05) | 0.10 (0.05) | 0.28 (0.18) | -0.23 (0.12) | -0.23 (0.17) |
| Med IncX2006 ^c^ | 0.26 (0.05) | 0.21 (0.05) | 0.54 (0.18) | -0.16 (0.10) | -0.05 (0.16) |
| Med IncX2009 ^c^ | 0.16 (0.05) | 0.15 (0.05) | 0.43 (0.17) | -0.16 (0.10) | -0.28 (0.18) |
| High IncX1993 ^c^ | -0.04 (0.04) | -0.04 (0.04) | 0.11 (0.28) | -- | -- |
| High IncX1997 ^c^ | 0.07 (0.04) | 0.06 (0.04) | 0.63 (0.22) | ref | ref |
| High IncX2000 ^c^ | 0.01 (0.04) | 0.02 (0.04) | 0.55 (0.20) | 0.01 (0.11) | -0.14 (0.14) |
| High IncX2004 ^c^ | 0.10 (0.06) | 0.08 (0.06) | 0.20 (0.20) | -0.36 (0.12) | -0.15 (0.16) |
| High IncX2006 ^c^ | 0.26 (0.06) | 0.21 (0.06) | 0.33 (0.19) | -0.33 (0.13) | 0.02 (0.16) |
| High IncX2009 ^c^ | 0.14 (0.06) | 0.14 (0.06) | 0.41 (0.18) | -0.22 (0.11) | -0.17 (0.18) |
| Med UrbXMed IncX1993^b,c^ | 0.03 (0.07) | 0.06 (0.07) | -0.29 (0.34) | -- | -- |
| Med UrbXMed IncX1997^b,c^ | -0.01 (0.06) | -0.03 (0.06) | -0.50 (0.28) | ref | -- |
| Med UrbXMed IncX2000^b,c^ | 0.06 (0.07) | 0.06 (0.07) | -0.75 (0.27) | -0.31 (0.17) | -- |
| Med UrbXMed IncX2004^b,c^ | -0.01 (0.08) | -0.02 (0.08) | -0.19 (0.27) | -0.08 (0.17) | -- |
| Med UrbXMed IncX2006^b,c^ | 0.00 (0.09) | -0.03 (0.09) | -0.44 (0.26) | -0.13 (0.16) | -- |
| Med UrbXMed IncX2009^b,c^ | 0.02 (0.09) | -0.03 (0.09) | -0.39 (0.25) | 0.14 (0.16) | -- |
| Med UrbXHigh IncX1993^b,c^ | 0.18 (0.07) | 0.20 (0.07) | -0.09 (0.37) | -- | -- |
| Med UrbXHigh IncX1997^b,c^ | -0.04 (0.07) | -0.08 (0.06) | -0.90 (0.30) | ref | -- |
| Med UrbXHigh IncX2000^b,c^ | 0.15 (0.07) | 0.10 (0.07) | -0.79 (0.29) | -0.40 (0.17) | -- |
| Med UrbXHigh IncX2004^b,c^ | 0.01 (0.09) | -0.03 (0.09) | 0.02 (0.27) | -0.05 (0.18) | -- |
| Med UrbXHigh IncX2006^b,c^ | -0.04 (0.09) | -0.10 (0.09) | -0.13 (0.27) | -0.38 (0.18) | -- |
| Med UrbXHigh IncX2009^b,c^ | 0.00 (0.09) | -0.06 (0.09) | -0.49 (0.26) | 0.07 (0.19) | -- |
| High UrbXMed IncX1993^b,c^ | -0.09 (0.07) | -0.07 (0.07) | -0.32 (0.31) | -- | -- |
| High UrbXMed IncX1997^b,c^ | 0.02 (0.08) | -0.14 (0.07) | -0.25 (0.25) | ref | -- |
| High UrbXMed IncX2000^b,c^ | 0.04 (0.08) | -0.09 (0.08) | -0.58 (0.26) | -0.12 (0.16) | -- |
| High UrbXMed IncX2004^b,c^ | -0.08 (0.10) | -0.13 (0.10) | -0.40 (0.25) | 0.14 (0.23) | -- |
| High UrbXMed IncX2006^b,c^ | -0.12 (0.11) | -0.23 (0.11) | -0.88 (0.25) | 0.25 (0.21) | -- |
| High UrbXMed IncX2009^b,c^ | 0.05 (0.09) | -0.17 (0.09) | -0.65 (0.24) | 0.07 (0.18) | -- |
| High UrbXHigh IncX1993^b,c^ | 0.09 (0.07) | 0.09 (0.07) | -0.04 (0.34) | -- | -- |
| High UrbXHigh IncX1997^b,c^ | 0.16 (0.08) | -0.04 (0.08) | -0.48 (0.27) | ref | -- |
| High UrbXHigh IncX2000^b,c^ | 0.23 (0.08) | -0.02 (0.08) | -0.54 (0.28) | -0.07 (0.16) | -- |
| High UrbXHigh IncX2004^b,c^ | 0.00 (0.10) | -0.25 (0.10) | -0.28 (0.26) | 0.44 (0.22) | -- |
| High UrbXHigh IncX2006^b,c^ | -0.16 (0.10) | -0.43 (0.10) | -0.55 (0.26) | 0.47 (0.19) | -- |
| High UrbXHigh IncX2009^b,c^ | 0.05 (0.10) | -0.24 (0.10) | -0.51 (0.24) | 0.20 (0.18) | -- |
| Age 18-35y^d^ | ref | ref | ref | ref | ref |
| Age 35-55y^d^ | 0.12 (0.01) | 0.05 (0.01) | 0.03 (0.04) | -0.04 (0.03) | -0.18 (0.05) |
| Age 55-75y^d^ | -0.16 (0.02) | -0.06 (0.01) | 0.26 (0.04) | -0.09 (0.03) | -0.11 (0.06) |
| North^e^ | ref | ref | ref | ref | ref |
| Central^e^ | -0.15 (0.02) | -0.14 (0.02) | -0.04 (0.03) | -0.01 (0.03) | -0.04 (0.06) |
| South^e^ | -0.08 (0.02) | -0.09 (0.02) | 0.00 (0.03) | -0.09 (0.03) | 0.05 (0.06) |
| Constant | 6.41 (0.02) | 6.39 (0.02) | 3.49 (0.10) | 2.36 (0.05) | 2.93 (0.12) |
| **Inflation Factors^f^** |  |  |  |  |  |
| 1991 | ref | ref | ref | -- | -- |
| 1993 | 0.43 (0.13) | 0.37 (0.11) | 0.52 (0.04) | -- | -- |
| 1997 | 0.76 (0.13) | 1.42 (0.10) | 0.47 (0.04) | ref | ref |
| 2000 | 0.78 (0.13) | 1.71 (0.10) | 0.29 (0.04) | 0.10 (0.04) | 0.11 (0.06) |
| 2004 | 0.14 (0.15) | 1.84 (0.10) | 0.01 (0.05) | 0.33 (0.05) | 0.30 (0.07) |
| 2006 | 0.60 (0.14) | 2.02 (0.10) | -0.04 (0.05) | 0.37 (0.05) | 0.26 (0.07) |
| 2009 | 1.43 (0.12) | 2.27 (0.10) | 0.00 (0.05) | 0.69 (0.04) | 0.29 (0.06) |
| Low Urb^b^ | ref | ref | ref | ref | ref |
| Medium Urb^b^ | 0.62 (0.09) | 0.91 (0.06) | -0.13 (0.04) | 0.23 (0.04) | -0.85 (0.07) |
| High Urb^b^ | 1.09 (0.08) | 2.08 (0.06) | -0.54 (0.04) | 0.48 (0.04) | -1.76 (0.07) |
| Low Inc^c^ | ref | ref | ref | ref | ref |
| Medium Inc^c^ | -0.56 (0.07) | -0.62 (0.05) | -0.02 (0.03) | -0.22 (0.04) | -0.27 (0.06) |
| High Inc^c^ | -0.74 (0.08) | -0.97 (0.05) | -0.01 (0.03) | -0.11 (0.04) | -0.57 (0.06) |
| Constant | -4.07 (0.12) | -4.15 (0.10) | -0.01 (0.04) | -0.62 (0.04) | 3.17 (0.08) |
| Ln(alpha)^g^ | -0.69 (0.01) | -0.97 (0.01) | 0.11 (0.02) | -0.41 (0.03) | -0.33 (0.03) |

^a^Separate zero-inflated negative binomial models estimated total, occupational, or domestic PA in 1991, 1993, 1997, 2000, 2004, 2006, and 2009. Travel and leisure PA were estimated in 1997, 2000, 2004, 2006, and 2009.

^b^Using individual and community-level data, a multicomponent urbanicity scale was created to represent the infrastructure, economic, and social service domains of the urban environment .[18] The scale has a possible score of 0-120 points, with a higher score representing more urban characteristics. The urbanicity scale was separated into year-specific tertiles: low, medium, and high urbanicity. Referent: low urbanicity

^c^Income defined by year-specific tertiles of household income. Referent: low income

^d^Age defined at each exam as young adult (18-35y), middle aged (35-55y), and old age (55-75y). Referent: young adult

^e^Region of China. Referent: North region

^f^Inflation factors are parameters included in a logistic regression model to predict whether or not a person is eligible to participate in the PA domain (i.e., a true zero).

^g^Alpha is the dispersion parameter which is used to determine if zero-inflation in the model is necessary.

Abbreviations: PA: physical activity; urb: urbanicity; inc: income

**Supplemental Table 2. Beta coefficients from zero-inflated negative binomial models in women^a^**

|  | **Total PA** | **Occupational PA** | **Domestic PA** | **Travel PA** | **Leisure PA** |
| --- | --- | --- | --- | --- | --- |
| Negative Binomial Parameter Estimates | |  |  |  |  |
| Low Urb^b^ | ref | ref | ref | ref | ref |
| Medium Urb^b^ | -0.31 (0.03) | -0.28 (0.03) | 0.08 (0.06) | 0.22 (0.08) | 0.00 (0.21) |
| High Urb^b^ | -1.06 (0.04) | -1.11 (0.04) | -0.05 (0.07) | 0.48 (0.07) | 0.31 (0.18) |
| Low Inc^c^ | ref | ref | ref | ref | ref |
| Medium Inc^c^ | -0.07 (0.02) | -0.06 (0.02) | -0.04 (0.06) | 0.17 (0.08) | -0.26 (0.14) |
| High Inc^c^ | -0.07 (0.03) | -0.05 (0.02) | 0.01 (0.06) | 0.11 (0.10) | -0.29 (0.14) |
| Med UrbXMed Inc^b,c^ | 0.09 (0.04) | 0.07 (0.04) | -0.07 (0.09) | -0.12 (0.12) | 0.00 (0.00) |
| Med UrbXHigh Inc^b,c^ | 0.05 (0.04) | 0.02 (0.04) | -0.07 (0.09) | -0.07 (0.12) | 0.00 (0.00) |
| High UrbXMed Inc^b,c^ | 0.12 (0.05) | 0.04 (0.05) | -0.07 (0.09) | -0.13 (0.11) | 0.00 (0.00) |
| High UrbXHigh Inc^b,c^ | 0.10 (0.06) | 0.03 (0.06) | -0.17 (0.10) | -0.10 (0.12) | 0.00 (0.00) |
| 1991 | ref | ref | ref | -- | -- |
| 1993 | -0.26 (0.02) | -0.25 (0.02) | -0.24 (0.05) | -- | -- |
| 1997 | -0.31 (0.02) | -0.25 (0.02) | -0.50 (0.05) | ref | ref |
| 2000 | -0.48 (0.02) | -0.42 (0.02) | -0.59 (0.05) | 0.18 (0.07) | 0.12 (0.28) |
| 2004 | -0.77 (0.04) | -0.67 (0.04) | -0.24 (0.06) | 0.04 (0.07) | 1.47 (0.31) |
| 2006 | -0.84 (0.04) | -0.72 (0.04) | -0.46 (0.06) | -0.14 (0.06) | 0.98 (0.30) |
| 2009 | -0.74 (0.04) | -0.68 (0.04) | -0.42 (0.05) | 0.01 (0.06) | 0.87 (0.26) |
| Med UrbX1993^b^ | 0.02 (0.04) | 0.03 (0.04) | -0.03 (0.08) | -- | -- |
| Med UrbX1997^b^ | -0.01 (0.04) | 0.08 (0.04) | -0.16 (0.07) | ref | ref |
| Med UrbX2000 ^b^ | -0.01 (0.05) | 0.08 (0.05) | -0.02 (0.07) | -0.02 (0.13) | 0.28 (0.30) |
| Med UrbX2004 ^b^ | -0.04 (0.06) | 0.04 (0.06) | -0.26 (0.09) | -0.46 (0.12) | -0.70 (0.33) |
| Med UrbX2006 ^b^ | 0.07 (0.06) | 0.14 (0.06) | 0.03 (0.09) | -0.25 (0.11) | -0.13 (0.34) |
| Med UrbX2009 ^b^ | -0.10 (0.06) | -0.03 (0.06) | -0.03 (0.08) | -0.26 (0.14) | -0.44 (0.28) |
| High UrbX1993 ^b^ | 0.18 (0.05) | 0.29 (0.05) | -0.03 (0.09) | -- | -- |
| High UrbX1997 ^b^ | -0.18 (0.06) | 0.05 (0.06) | 0.02 (0.08) | ref | ref |
| High UrbX2000 ^b^ | -0.23 (0.07) | 0.16 (0.07) | 0.02 (0.09) | -0.25 (0.10) | 0.10 (0.25) |
| High UrbX2004 ^b^ | -0.03 (0.07) | 0.30 (0.08) | -0.07 (0.09) | -1.01 (0.15) | -1.02 (0.30) |
| High UrbX2006 ^b^ | 0.12 (0.07) | 0.44 (0.08) | 0.16 (0.09) | -0.76 (0.13) | -0.64 (0.29) |
| High UrbX2009 ^b^ | -0.03 (0.07) | 0.36 (0.09) | 0.15 (0.09) | -0.65 (0.14) | -0.63 (0.25) |
| Med IncX1993^c^ | 0.07 (0.03) | 0.06 (0.03) | 0.17 (0.09) | -- | -- |
| Med IncX1997 ^c^ | 0.07 (0.03) | 0.08 (0.03) | -0.06 (0.07) | ref | ref |
| Med IncX2000 ^c^ | 0.10 (0.04) | 0.06 (0.04) | 0.14 (0.08) | -0.09 (0.12) | 0.06 (0.20) |
| Med IncX2004 ^c^ | 0.24 (0.05) | 0.13 (0.05) | -0.02 (0.09) | -0.29 (0.12) | 0.21 (0.19) |
| Med IncX2006 ^c^ | 0.25 (0.05) | 0.19 (0.05) | 0.12 (0.09) | -0.15 (0.10) | 0.08 (0.22) |
| Med IncX2009 ^c^ | 0.22 (0.05) | 0.15 (0.06) | 0.21 (0.08) | -0.10 (0.12) | 0.43 (0.17) |
| High IncX1993 ^c^ | 0.01 (0.04) | -0.01 (0.04) | 0.14 (0.10) | -- | -- |
| High IncX1997 ^c^ | 0.07 (0.04) | 0.06 (0.04) | -0.14 (0.08) | ref | ref |
| High IncX2000 ^c^ | 0.15 (0.04) | 0.10 (0.04) | 0.06 (0.08) | -0.08 (0.12) | -0.22 (0.21) |
| High IncX2004 ^c^ | 0.17 (0.06) | 0.09 (0.06) | -0.01 (0.10) | -0.35 (0.12) | 0.14 (0.18) |
| High IncX2006 ^c^ | 0.25 (0.06) | 0.18 (0.06) | 0.11 (0.10) | -0.32 (0.13) | 0.24 (0.22) |
| High IncX2009 ^c^ | 0.17 (0.06) | 0.07 (0.06) | 0.21 (0.09) | -0.21 (0.12) | 0.22 (0.17) |
| Med UrbXMed IncX1993^b,c^ | -0.03 (0.06) | -0.01 (0.06) | -0.06 (0.13) | -- | -- |
| Med UrbXMed IncX1997^b,c^ | 0.02 (0.06) | -0.05 (0.06) | 0.19 (0.11) | ref | -- |
| Med UrbXMed IncX2000^b,c^ | -0.07 (0.07) | -0.07 (0.07) | -0.05 (0.12) | 0.07 (0.18) | -- |
| Med UrbXMed IncX2004^b,c^ | -0.08 (0.08) | -0.08 (0.09) | 0.20 (0.12) | 0.06 (0.17) | -- |
| Med UrbXMed IncX2006^b,c^ | -0.04 (0.09) | -0.06 (0.09) | -0.01 (0.13) | 0.00 (0.16) | -- |
| Med UrbXMed IncX2009^b,c^ | -0.10 (0.09) | -0.07 (0.09) | 0.01 (0.12) | -0.15 (0.19) | -- |
| Med UrbXHigh IncX1993^b,c^ | 0.13 (0.06) | 0.16 (0.06) | -0.11 (0.13) | -- | -- |
| Med UrbXHigh IncX1997^b,c^ | -0.02 (0.06) | -0.06 (0.06) | 0.16 (0.11) | ref | -- |
| Med UrbXHigh IncX2000^b,c^ | 0.01 (0.07) | -0.01 (0.07) | 0.03 (0.12) | 0.13 (0.18) | -- |
| Med UrbXHigh IncX2004^b,c^ | 0.05 (0.09) | 0.02 (0.09) | 0.19 (0.13) | 0.21 (0.17) | -- |
| Med UrbXHigh IncX2006^b,c^ | -0.03 (0.09) | -0.07 (0.09) | -0.05 (0.14) | 0.29 (0.20) | -- |
| Med UrbXHigh IncX2009^b,c^ | 0.00 (0.09) | 0.06 (0.09) | -0.02 (0.13) | -0.02 (0.19) | -- |
| High UrbXMed IncX1993^b,c^ | -0.06 (0.07) | -0.09 (0.07) | -0.04 (0.13) | -- | -- |
| High UrbXMed IncX1997^b,c^ | 0.02 (0.08) | -0.10 (0.08) | 0.15 (0.11) | ref | -- |
| High UrbXMed IncX2000^b,c^ | 0.01 (0.08) | -0.19 (0.09) | 0.01 (0.12) | 0.15 (0.15) | -- |
| High UrbXMed IncX2004^b,c^ | -0.11 (0.09) | -0.09 (0.11) | 0.12 (0.13) | 0.33 (0.21) | -- |
| High UrbXMed IncX2006^b,c^ | -0.24 (0.09) | -0.19 (0.11) | -0.10 (0.13) | 0.41 (0.17) | -- |
| High UrbXMed IncX2009^b,c^ | -0.07 (0.09) | -0.20 (0.11) | -0.07 (0.12) | 0.17 (0.21) | -- |
| High UrbXHigh IncX1993^b,c^ | 0.05 (0.07) | 0.00 (0.07) | -0.05 (0.14) | -- | -- |
| High UrbXHigh IncX1997^b,c^ | 0.16 (0.08) | 0.04 (0.08) | 0.17 (0.12) | ref | -- |
| High UrbXHigh IncX2000^b,c^ | 0.06 (0.08) | -0.16 (0.09) | 0.08 (0.13) | 0.13 (0.15) | -- |
| High UrbXHigh IncX2004^b,c^ | 0.07 (0.09) | -0.10 (0.11) | 0.14 (0.14) | 0.62 (0.20) | -- |
| High UrbXHigh IncX2006^b,c^ | -0.14 (0.09) | -0.33 (0.11) | -0.01 (0.14) | 0.55 (0.18) | -- |
| High UrbXHigh IncX2009^b,c^ | 0.05 (0.09) | -0.18 (0.11) | -0.04 (0.13) | 0.25 (0.19) | -- |
| Age 18-35y^d^ | ref | ref | ref | ref | ref |
| Age 35-55y^d^ | 0.16 (0.01) | 0.15 (0.01) | -0.16 (0.02) | -0.05 (0.02) | 0.06 (0.06) |
| Age 55-75y^d^ | -0.26 (0.02) | -0.04 (0.02) | -0.13 (0.02) | -0.23 (0.04) | 0.19 (0.07) |
| North^e^ | ref | ref | ref | ref | ref |
| Central^e^ | -0.09 (0.02) | -0.11 (0.02) | 0.01 (0.02) | 0.03 (0.03) | 0.11 (0.07) |
| South^e^ | 0.03 (0.02) | -0.01 (0.02) | -0.02 (0.02) | -0.03 (0.03) | 0.24 (0.07) |
| Constant | 6.45 (0.02) | 6.33 (0.02) | 4.43 (0.04) | 2.20 (0.05) | 2.93 (0.21) |
| **Inflation Factors^f^** |  |  |  |  |  |
| 1991 | ref | ref | ref | -- | -- |
| 1993 | 0.42 (0.18) | 0.11 (0.07) | 0.46 (0.07) | -- | -- |
| 1997 | 0.80 (0.16) | 1.06 (0.07) | 0.68 (0.07) | ref | ref |
| 2000 | 0.93 (0.16) | 1.37 (0.07) | 0.51 (0.07) | 0.06 (0.04) | 0.14 (0.08) |
| 2004 | 0.94 (0.16) | 1.90 (0.07) | 0.20 (0.08) | 0.73 (0.04) | -0.15 (0.08) |
| 2006 | 0.96 (0.16) | 2.01 (0.07) | 0.04 (0.08) | 0.75 (0.04) | -0.24 (0.08) |
| 2009 | 0.85 (0.17) | 2.05 (0.07) | -0.14 (0.08) | 0.92 (0.04) | -0.34 (0.08) |
| Low Urb^b^ | ref | ref | ref | ref | ref |
| Medium Urb^b^ | 0.50 (0.10) | 0.98 (0.05) | 0.03 (0.05) | 0.23 (0.04) | -1.27 (0.11) |
| High Urb^b^ | 0.73 (0.10) | 2.16 (0.05) | 0.09 (0.05) | 0.58 (0.04) | -2.43 (0.11) |
| Low Inc^c^ | ref | ref | ref | ref | ref |
| Medium Inc^c^ | -0.29 (0.09) | -0.60 (0.04) | -0.02 (0.05) | -0.34 (0.04) | -0.41 (0.07) |
| High Inc^c^ | -0.57 (0.09) | -0.87 (0.04) | 0.15 (0.05) | -0.39 (0.04) | -0.54 (0.07) |
| Constant | -4.59 (0.15) | -3.35 (0.07) | -2.56 (0.07) | -0.57 (0.05) | 4.65 (0.12) |
| Ln(alpha)^g^ | -0.65 (0.01) | -0.87 (0.01) | -0.56 (0.01) | -0.53 (0.03) | -0.40 (0.04) |

^a^Separate zero-inflated negative binomial models were used to estimated total, occupational, or domestic PA in 1991, 1993, 1997, 2000, 2004, 2006, and 2009. Travel and leisure PA were estimated in 1997, 2000, 2004, 2006, and 2009.

^b^Using individual and community-level data, a multicomponent urbanicity scale was created to represent the infrastructure, economic, and social service domains of the urban environment [18] The scale has a possible score of 0-120 points, with a higher score representing more urban characteristics. The urbanicity scale was separated into year-specific tertiles: low, medium, and high urbanicity. Referent: low urbanicity

^c^Income defined by year-specific tertiles of household income. Referent: low income

^d^Age defined at each exam as young adult (18-35y), middle aged (35-55y), and old age (55-75y). Referent: young adult

^e^Region of China. Referent: North region

^f^Inflation factors are parameters included in a logistic regression model to predict whether or not a person is eligible to participate in the PA domain (i.e., a true zero).

^g^Alpha is the dispersion parameter which is used to determine if zero-inflation in the model is necessary.

Abbreviations: PA: physical activity; urb: urbanicity; inc: income

**Supplemental Figure 2. Predicted mean occupational PA, China Health and Nutrition Survey 1991-2009^a^**


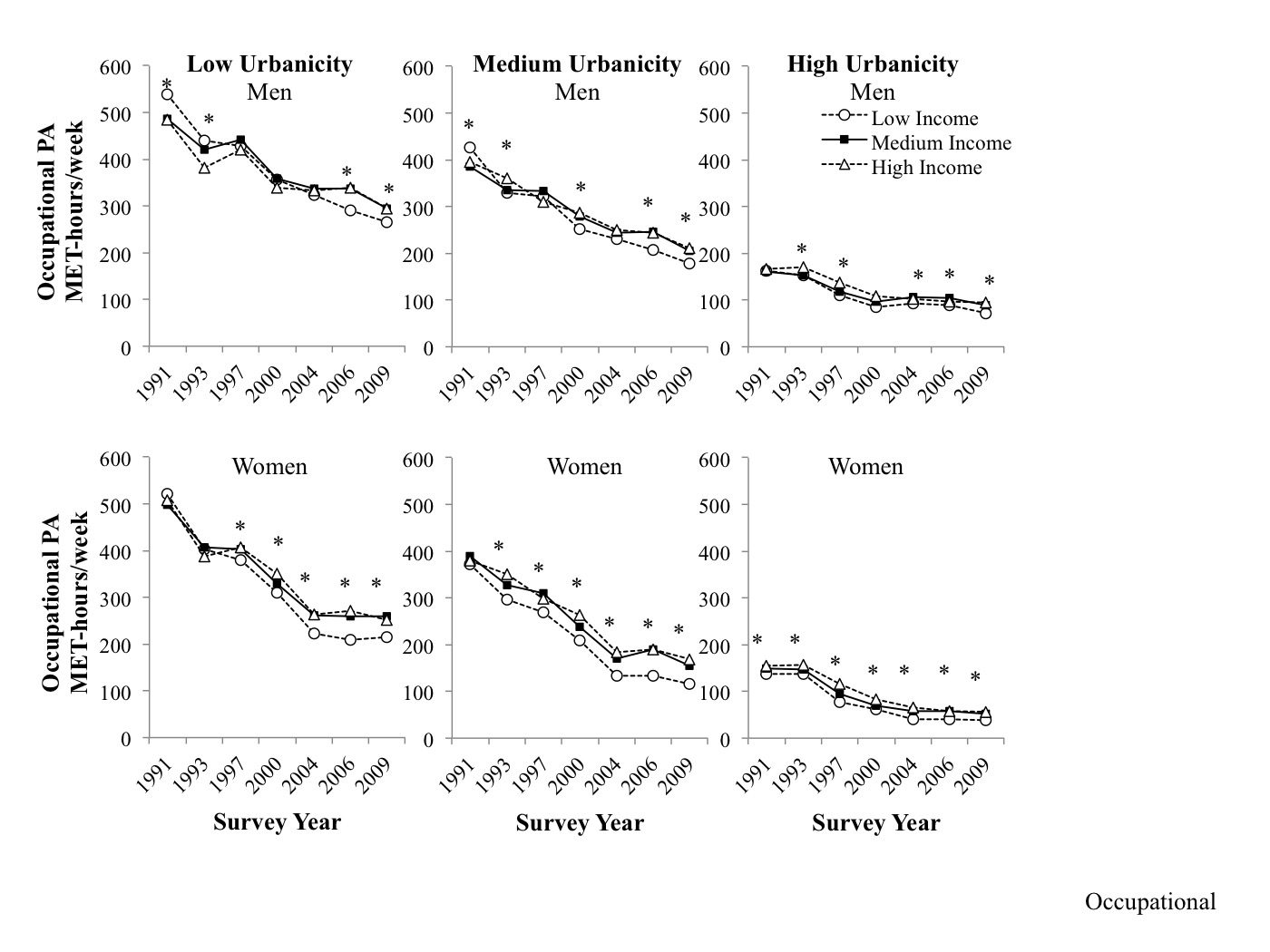


^a^Adjusted mean occupational physical activity predicted from sex-stratified, zero-inflated negative binomial models according to urbanicity (year-specific tertiles of low, medium, and high urbanicity) and income (year-specific tertiles of low, medium, and high income). Main exposure variables were year, urbanicity, income, the interaction between urbanicity and income, and the interaction between urbanicity, income and year. Models additionally control for region of China (North, Central, South) and age (ages 18-35y, 35-55y, 55-75y). Stars denote a statistically significant difference in mean PA for high versus low income at the p<0.05 level for at each s year.

Abbreviations: PA, physical activity.

**Supplemental Figure 3. Predicted domestic PA, China Health and Nutrition Survey 1991-2009^a^**


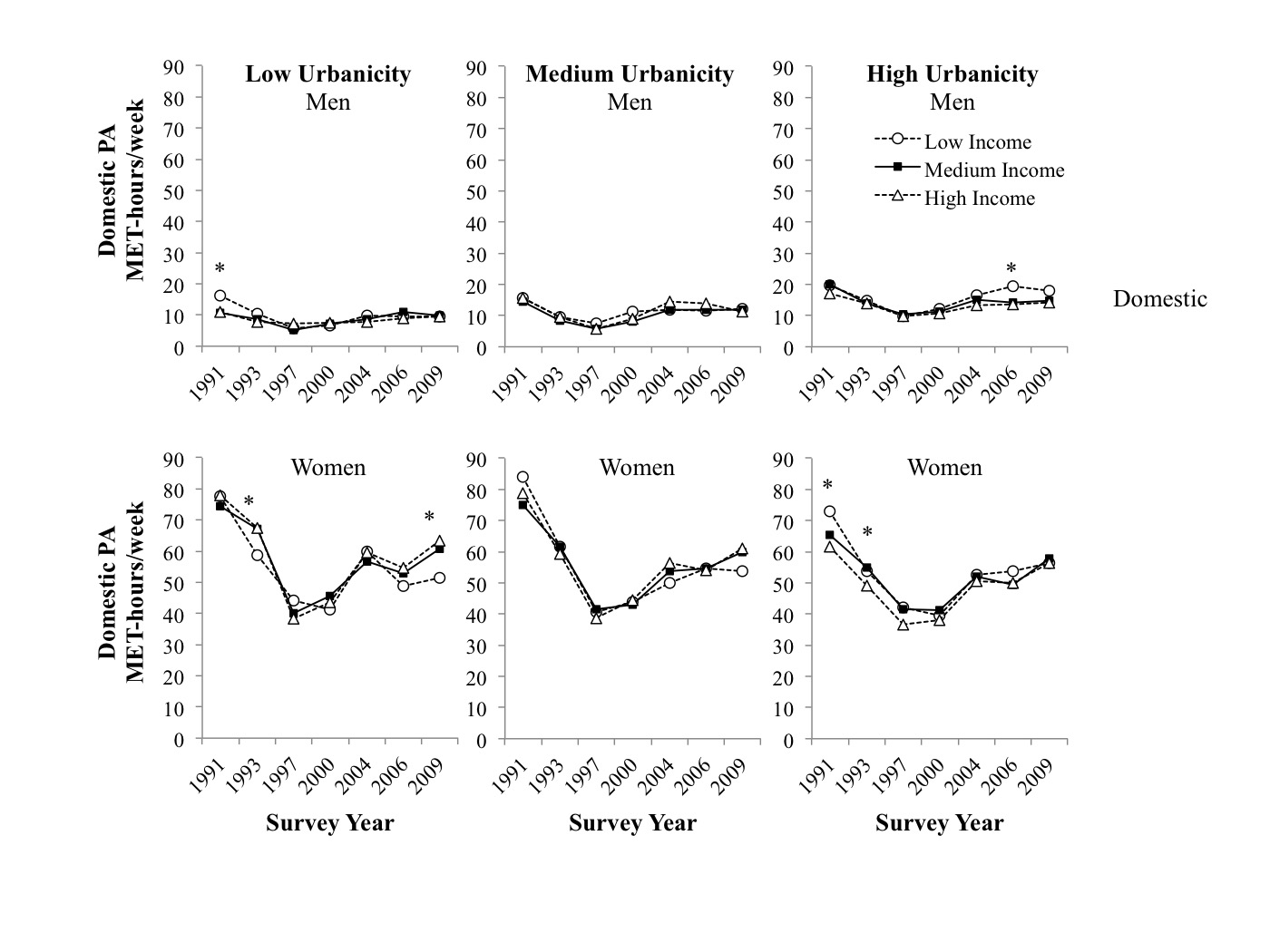


^a^Adjusted mean domestic physical activity predicted from sex-stratified, zero-inflated negative binomial models according to urbanicity (year-specific tertiles of low, medium, and high urbanicity) and income (year-specific tertiles of low, medium, and high income). Main exposure variables were year, urbanicity, income, the interaction between urbanicity and income, and the interaction between urbanicity, income and year. Models additionally control for region of China (North, Central, South) and age (ages 18-35y, 35-55y, 55-75y). Stars denote a statistically significant difference in mean PA for high versus low income at the p<0.05 level for at each year.

Abbreviations: PA, physical activity.

**Supplemental Figure 4. Predicted mean travel PA, China Health and Nutrition Survey 1991-2009^a^**


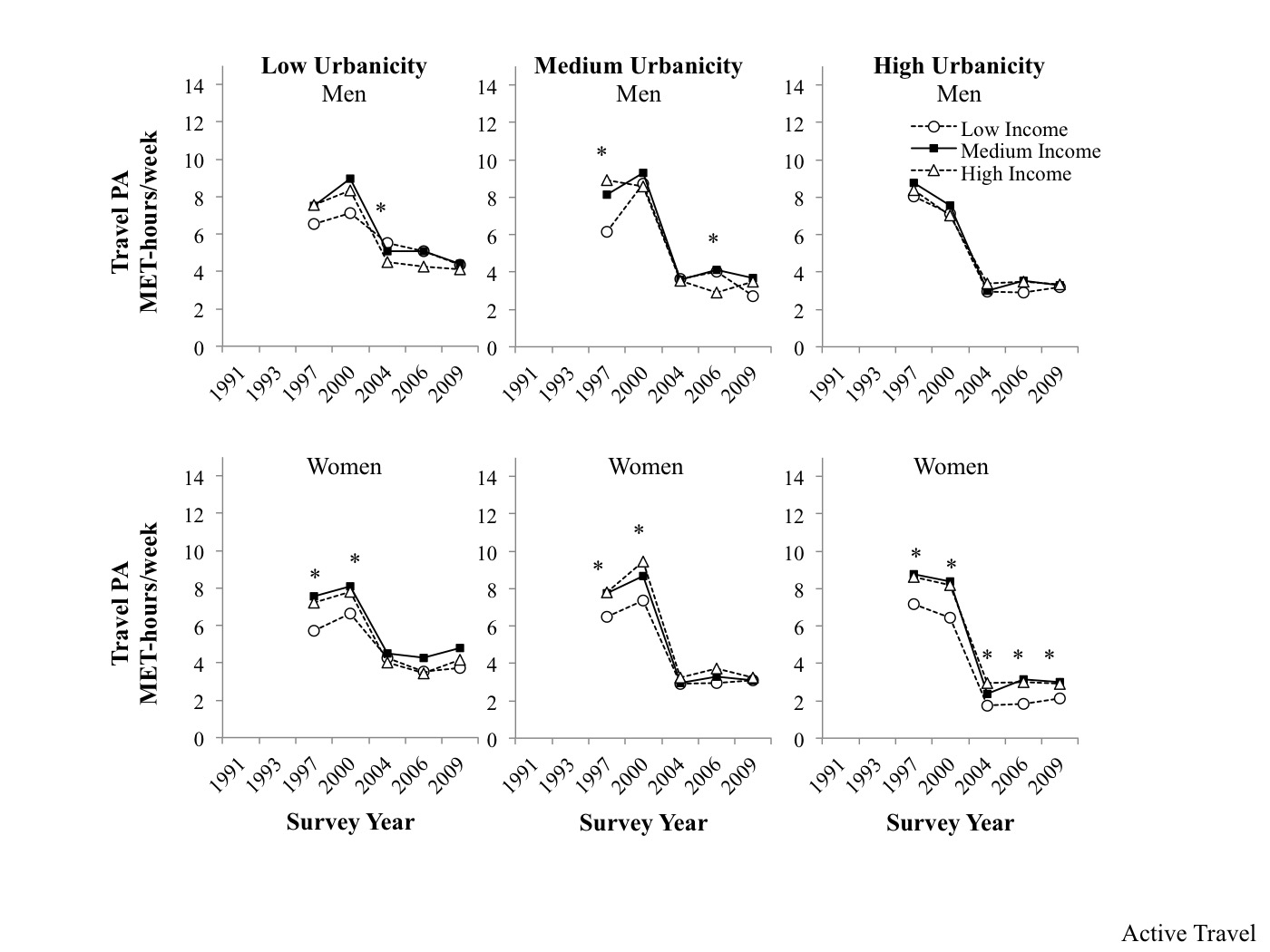


^a^Adjusted mean travel physical activity predicted from sex-stratified, zero-inflated negative binomial models according to urbanicity (year-specific tertiles of low, medium, and high urbanicity) and income (year-specific tertiles of low, medium, and high income). Main exposure variables were year, urbanicity, income, the interaction between urbanicity and income, and the interaction between urbanicity, income and year. Models additionally control for region of China (North, Central, South) and age (ages 18-35y, 35-55y, 55-75y). Stars denote a statistically significant difference in mean PA for high versus low income at the p<0.05 level for at each year.

Abbreviations: PA, physical activity.

**Supplemental Figure 5. Predicted mean leisure PA, China Health and Nutrition Survey 1991-2009^a^**


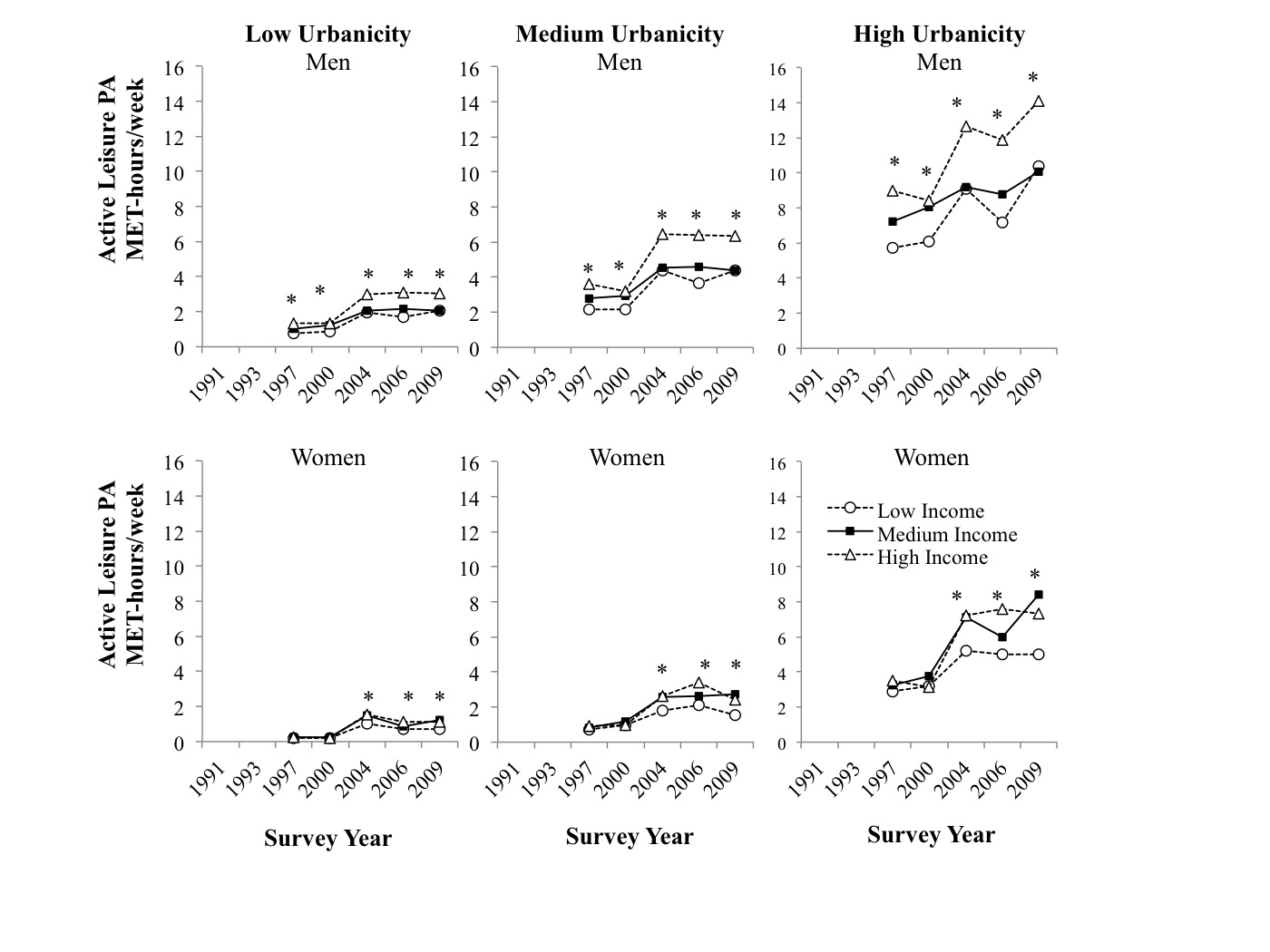


^a^Adjusted mean leisure physical activity predicted from sex-stratified, zero-inflated negative binomial models according to urbanicity (syear-specific tertiles of low, medium, and high urbanicity) and income (year-specific tertiles of low, medium, and high income). Main exposure variables were year, urbanicity, income, the interaction between urbanicity and income, and the interaction between urbanicity, income and year. Models additionally control for region of China (North, Central, South) and age (ages 18-35y, 35-55y, 55-75y). Stars denote a statistically significant difference in mean PA for high versus low income at the p<0.05 level for at each year.

Abbreviations: PA, physical activity.

**Supplemental Figure 6. Predicted mean sedentary PA^a^**


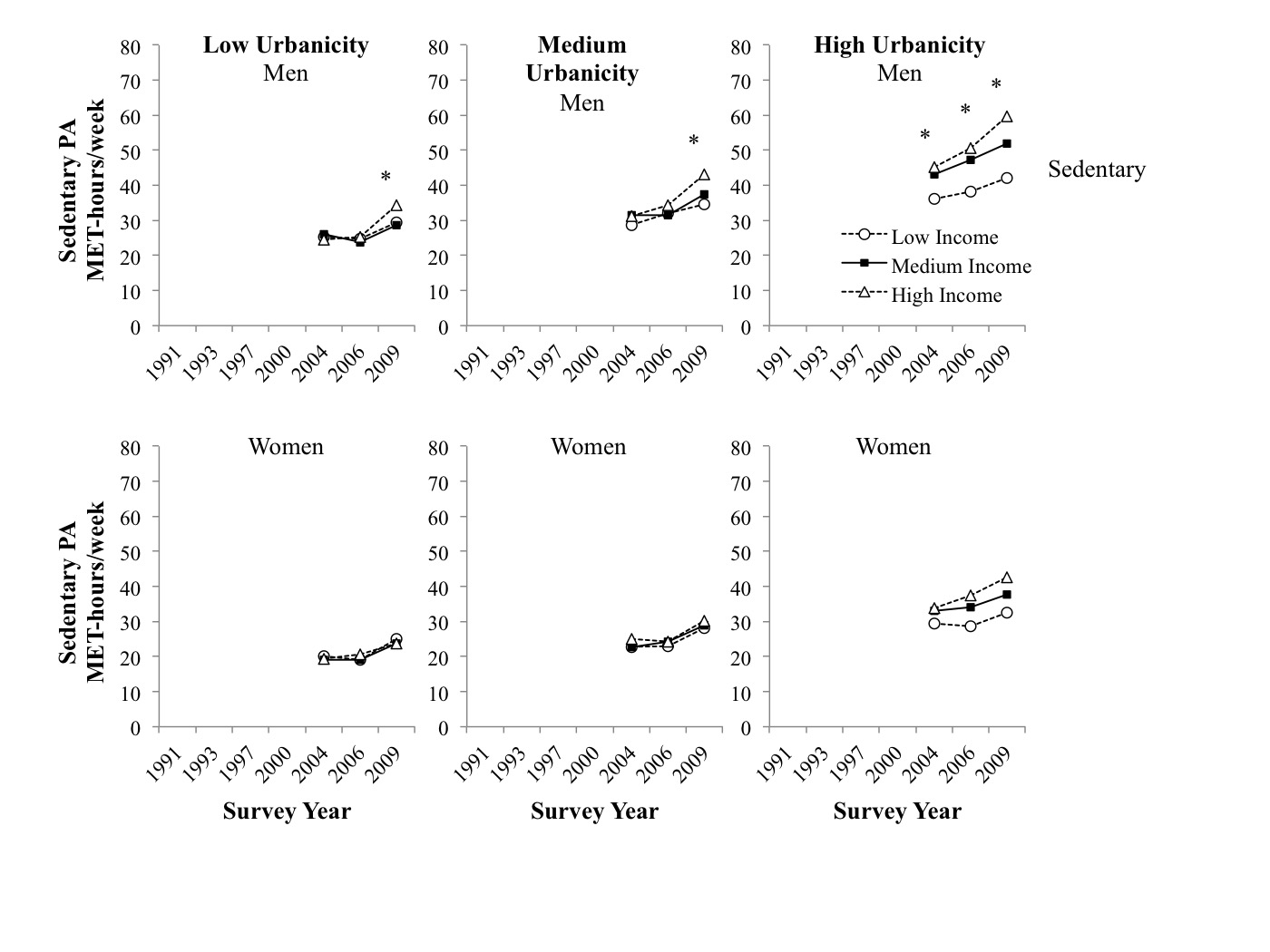


^a^Adjusted mean sedentary physical activity from sex-stratified, zero-inflated negative binomial models according to urbanicity (year-specific tertiles of low, medium, and high urbanicity) and income (year-specific tertiles of low, medium, and high income). Main exposure variables were year, urbanicity, income, the interaction between urbanicity and income, and the interaction between urbanicity, income and year. Models additionally control for region of China (North, Central, South) and age (ages 18-35y, 35-55y, 55-75y). Stars denote a statistically significant difference in mean PA for high versus low income at the p<0.05 level for at each year.

Abbreviations: PA, physical activity.
